# Association of self-efficacy and mental toughness with sport performance in Brazilian futsal athletes

**DOI:** 10.3389/fpsyg.2023.1195721

**Published:** 2023-07-21

**Authors:** Paulo Vitor Suto Aizava, Renan Codonhato, Lenamar Fiorese

**Affiliations:** Sports Psychology and Human Performance Study Group, Physical Education Department, Maringá State University, Maringá, Brazil

**Keywords:** sport self-efficacy, performance, futsal, mental toughness, Brazilian athletes

## Abstract

**Introduction:**

Self-efficacy is considered a component of mental toughness, but there are few studies investigating the relationship of sport self-efficacy with mental toughness in performance athletes, especially in team sports.

**Objective:**

The objective was to examine the impact of sport self-efficacy mediated by mental toughness on the sport performance of Brazilian futsal athletes.

**Methods:**

The sample was composed of five adult male teams participating in the National Futsal League 2020, totaling 77 athletes. As instruments, we used: athlete identification sheet, Perceived Self-Efficacy Scale in Sports (PSES), Mental Toughness Index (MTI) questionnaire and the performance data from the National Futsal League 2020 (NFL). The data were analyzed using the Kolmogorov–Smirnov test, Mann–Whitney “U” test, Spearman’s correlation, network analysis (LASSO), with the indicators of centrality: strength, proximity and degree of intermediation (*p* < 0.05).

**Results:**

The results showed that the investigated sample presented high levels of Perceived Self-Efficacy Scale in Sports (PSES) (Md = 4.66) and mental toughness (MT) (Md = 6.44). PSES presented a positive relationship with the number of wins, and negative relationships with the number of red cards and wrong passes (*r* = −0.08). MT indirectly influenced these variables through its connection with PSES (*r* = 0.30). The best ranked teams presented higher amounts of goals for, fouls, shots on goal, tackles, assists, and wins. The time of practice revealed an inverse relationship with the number of defeats, while age was positively related to the number of wrong passes (*r* = 0.09). The centrality indicators showed that the number of games stood out as the most central variable in the network, due to its degree of strength, proximity and intermediation. Moreover, the high degree of proximity and intermediation of the tackles made presented a connection with the number of assists (*γ* = 0.25; *n* = 77).

**Conclusion:**

We can conclude that sport self-efficacy and mental toughness are intervening factors in the sport performance of Brazilian futsal athletes.

## Introduction

Self-efficacy (SE) plays a key role in terms of understanding the dynamics and performance of sport teams (Jowett et al., 2012), in that it influences athletes’ decision-making, motivation, sport engagement, as well as collective performance and the way in which they will deal with sport failures (Alves et al., 2021). SE is considered a component of mental toughness (MT) (Crampton, 2014; Whitton et al., 2020), but there are few studies investigating the relationship of sport self-efficacy with mental toughness in performance athletes, especially in team sports.

Recent research has investigated SE in relation to quality of life in volleyball athletes (Aizava et al., 2021); systematic review on collective efficacy in soccer teams (Alves et al., 2021); confidence in using apps for health and sport (Chamorro-Koc et al., 2021); competitive cognitive anxiety, motor performance and goal orientations in college basketball (Peng and Zhang, 2021); health-related behavior in Taekwon-Do (Ortenburger et al., 2021). Accordingly, the present study aims to explore this gap by investigating the impact of self-efficacy mediated by mental toughness on the sport performance of Brazilian futsal athletes.

Until this moment, we have found two studies investigating both variables in ultramarathon runners (Brace et al., 2020), while Ramolale et al. (2021) examined the mediating role of MT in the relationship between self-efficacy and pro-social/antisocial behaviors in young athletes in Botswana. The authors pointed out the need to advance these investigations, highlighting a paucity of studies relating self-efficacy to mental toughness.

In this regard, Bandura et al. (2008) sought to explain the perception (belief) of SE through the Social Cognitive Theory (SCT) and the Triadic Reciprocal Model (TRM), in which thinking and behavior are products of the dynamic and reciprocal interrelationship between personal, behavioral and environmental influences. This perception determines the judgment of the ability of individuals to perform a certain task, directly influencing the results that these people expect to achieve with the joint actions (Bandura, 1977).

In the context of performance athletes, MT has an important role for good levels of sports performance, helping to deal with adverse situations: coping with stress, frustrations, challenges and everyday adversities, being essential for sporting success (Moreira et al., 2021). According to Gucciardi (2017), considering that MT is a dynamic and adaptive psychological resource, the investigation of this variable in athletes is essential, making it possible to evaluate clear objectives with efficiency in terms of developing and maintaining good decision-making (Anthony et al., 2020).

As hypotheses, it is expected that athletes present a good perception of sports self-efficacy and mental toughness, and that there is a good association between these variables, in addition to interactions with sports performance. Thus, the objective of this study was to examine the impact of sport self-efficacy mediated by mental toughness on the sport performance of Brazilian futsal athletes.

## Materials and methods

### Population and sample

The target population was composed of 252 athletes from the National Futsal League 2020 (NFL 2020). This competition was chosen because it is the main futsal competition in Brazil. Currently, the Brazilian men’s futsal team is the best team in the world in the world ranking of futsal teams, according to the Fédération Internationale de Football Association (FIFA, 2022), making the National Futsal League one of the most important leagues around the world.

As inclusion criteria were considered: being over 18 years old, having active and regular contracts in the teams and participating in the league. The following were excluded: athletes under the age of 18, athletes who were not participating in the league (due to contractual or injury reasons), athletes on leave due to Covid.

The sample was composed of 77 athletes with a mean age of 27.6 years (±4.6), who consented to participate in the study, belonging to five teams (each team had between 14 and 16 athletes). Due to the paralysis of the NFL 2020 as a result of the COVID-19 pandemic, all teams reported being with a reduced cast in relation to the original cast, because of the termination of contract of some athletes and consequent spending cuts.

### Instruments

The instruments used were the athlete identification sheet, the Perceived Self-Efficacy Scale in Sports (PSES), the Mental Toughness Index (MTI) questionnaire and the performance data made available on the National Futsal League (NFL) 2020 website.

### Athlete identification sheet

We used an identification sheet to record the athletes’ personal data such as age, position, time of practice and forms of contact (*e-mail, Whatsapp*).

### Perceived self-efficacy scale in sports

The Physical Education Efficacy Perceptions (PEEP) was initially developed by Jackson et al. (2012). This instrument is part of a series of analyses called Tripartite Efficacy Beliefs (TEB).

Three instruments were developed to evaluate perception and self-efficacy: self-efficacy perception (self-efficacy), athletes’ confidence in coaches’ efficacy (other-efficacy) and estimation of coaches’ confidence in their abilities (relation-inferred self-efficacy—RISE). The present study addressed the instrument that evaluates self-efficacy in itself, with adaptation and validity evidence for the sport context.

After the process of cross-cultural adaptation and validity evidence (Aizava, 2022), we observed that the Perceived Self-Efficacy Scale in Sports (PSES) presented good psychometric properties for its use in the sport context and all items were maintained. The nine questions are answered on a 5-point Likert scale: ranging from 1 (no confidence), 2 (low confidence), 3 (moderate confidence), 4 (high confidence), and 5 (total confidence), and their result is unifactorial through the overall mean of the questions (1–5).

Strong reliability was shown with Cronbach’s Alpha values, all above *α* > 0.70. Through the analysis of factorial loads obtained in the final model, the Composite Reliability (CR) obtained satisfactory values (CR = 0.87), and the Mean Extracted Variance (MEV) presented values within the limits recommended by the literature (MEV = 0.44). The instrument presented a value of *α* = 0.791, showing strong data reliability for the present study.

### Mental toughness index

In order to evaluate mental toughness, we applied the Mental Toughness Scale (MRS) initially developed by Gucciardi (2012) for the English language and validated for the Brazilian context by Moreira et al. (2021).

The scale contains eight questions about how the athlete generally thinks, feels and behaves during a sport practice, answered on a Likert scale from 1 to 7, and its result is given in a unidimensional form from the overall mean of the questions.

Mental toughness (MR) was rated at: 1 to 2—low; 3 to 4—medium; 5 to 7—high. The instrument showed a value of *α* = 0.764, demonstrating strong data reliability for the present study.

### Performance data—National Futsal League 2020

The athletes’ sports performance was obtained after the end of the NFL 2020. The data was collected through the website https://ligafutsal.com.br/estatisticas/, with information such as: final ranking, goals scored, fouls committed, fouls conceded, cards received, assists, tackles, and defenses.

### Data collection

The present research is part of the institutional project called “Development process of positive psychological variables in the sport context” (Opinion no 4.022.246). Considering that the collection took place during the pandemic period of COVID-19, we adopted a biosafety protocol with some precautionary and preventive measures, following the recommendations of the World Health Organization (WHO) for face-to-face collections:

- Prior scheduling and authorization from the teams’ supervisors, in a suitable place and time, avoiding crowds and wearing masks.
- Hand sanitization with alcohol gel 70° INPM for all research participants, before and after completing the questionnaires, as well as for the researcher.
- All questionnaires and pens were previously sanitized with alcohol 70° INPM and placed in individual plastic envelopes.
- The individual envelopes were placed on a table, or directly on the tables where each athlete would sit, so that each athlete could take his/her questionnaire, avoiding direct contact with the researcher and maintaining a distance of 2 m.
- After the collections, all pens were placed in a separate container for immediate sanitization with alcohol 70° INPM.
- All questionnaires (paper) were placed in a separate envelope and handled only after 5 days. After this period, the envelope was discarded and the questionnaires were stored in a new envelope.

Some questionnaires were sent via e-mail, completed, scanned and sent back to the researcher; other questionnaires were sent via e-mail, and later the researcher picked them up in person with the supervisor/technical coordinator. The data were collected individually, during the second semester of 2020, according to the availability of the athletes by completing the Free and Informed Consent Form (FICF).

### Data analysis

Data were evaluated using descriptive statistics (median and interquartile range) and inferential statistics through specific statistical parameters established by the Kolmogorov–Smirnov normality test (*n* > 50), in which the data presented a non-parametric distribution. Cronbach’s Alpha coefficient was used to verify data reliability, in addition to Mann–Whitney’s “U” test, Spearman’s correlation coefficient and network analyses (*p* < 0.05).

In order to verify the multivariate relationships between the investigated variables, the network analysis method was used. From the previously calculated Spearman’s correlation matrix, regularized networks of partial correlations called LASSO networks (Least Absolute Shrinkage and Selection Operator) were computed. LASSO regularization is a method used to control the presence of spurious correlations by reducing the partial correlation coefficients to zero, removing weak and trivial connections, and favoring simpler and more objective networks to be interpreted (Epskamp and Fried, 2018).

In order to configure the network selection, it is possible to adjust the hyperparameter *γ* (gamma). Higher values of gamma (*γ* = 0.5) result in simpler networks, with fewer connections and greater parsimony, also called a sparse network, which means that spurious correlations (false positive) will hardly be preserved; however, there is the possibility that true connections are also lost (false negative); on the other hand, lower values of gamma (*γ* = 0) favor the discovery of connections, although it requires greater caution when interpreting them (Epskamp and Fried, 2018). In this regard, three network models were computed with increasing values of gamma, from the least to the most conservative: Network 1 with *γ* = 0, Network 2 with *γ* = 0.25 and Network 3 with *γ* = 0.5. For the present study, we consider the network with *γ* = 0.25, as it is considered a more balanced network in its connections.

In general, networks are formed by “nodes” (circles representing variables) connected by edges, whose color and thickness vary according to the direction and intensity of the relationship between these variables, and the distribution and proximity of the nodes also correspond to the associations within the network (Silva et al., 2006). In order to identify the most influential or most important variables, we used the following network centrality indicators (NCI): Strength, which evaluates the sum of the weights/coefficients of each node; Proximity (Closeness), which evaluates the distance (length of the edges) connecting a node with the others and indicates the speed with which its information propagates in the network; and degree of intermediation (Betweenness), which represents the number of times a node acts as a bridge/connector on the shortest path between two nodes, that is, how much information passes through that variable within a network, indicating its potential to affect the other variables (Dalege et al., 2017).

## Results

The descriptive analyses allowed us to observe that the investigated futsal athletes had good levels of Perceived Self-Efficacy Scale in Sports (PSES) and Mental Toughness (MT) (Table 1).

**Table 1:** Descriptive analysis of perceived self-efficacy scale in sports (PSES), mental toughness (MT) and performance data of NFL 2020 teams from Paraná (*n* = 77).

	PSES		MT		G. F.	Y. C.	R. C.	F	S. G.	T.S.	A	W	D	D	G	F. R.
	Md	(Q1–Q3)	Md	(Q1–Q3)												
Team 1	4.60	(3.92–4.80)	6.50	(5.82–6.80)	51	46	1	124	211	1,145	28	8	3	5	16	5°
Team 2	4.65	(4.32–4.77)	6.40	(6.15–6.75)	28	42	4	119	218	1,084	15	4	8	4	16	7°
Team 3	4.90	(4.67–5.00)	6.70	(6.22–6.85)	31	28	2	101	168	1,005	23	5	5	4	14	9°
Team 4	4.30	(4.20–4.60)	6.10	(5.90–6.50)	22	27	1	93	157	957	14	3	5	6	14	16°
Team 5	4.85	(4.50–4.92)	6.50	(6.10–6.82)	24	24	2	74	130	748	7	4	0	8	12	17°

PSES, perceived self-efficacy ecale in sports; MT, mental toughness; G. P., goals for; Y. C., yellow cards; R. C., red cards; F, fouls; S.G., shots on goal; TS, tackles; A, assists; W, wins; D, draws; D, defeats; G, games; F. R., final ranking.

Considering that PSES varies between 1 and 5 and MT varies between 1 and 7, PSES was rated as: 1 to 2—low; 3—mean; 4 to 5—high; MT was evaluated in: 1 to 2—low; 3 to 4—mean; 5 to 7—high. Table 1 shows that all teams had excellent levels of PSES and MT, with median PSES above Md = 4.30 and median MT above Md = 6.10.

It can be seen that the best ranked teams (teams 1, 2, and 3) had higher amounts of goals for (GF), fouls (F), shots on goal (SG), tackles (TS), assists (A) and wins (W). Teams 1 and 2 received the greatest numbers of yellow cards (YC), with 46 and 42, respectively. Team 1 received only one red card (RC), while team 2 received four red cards. On the other hand, teams 4 and 5, with the worst final ranking (FR), presented worse indexes in the same observed performance variables (Table 1).

**Figure 1:**
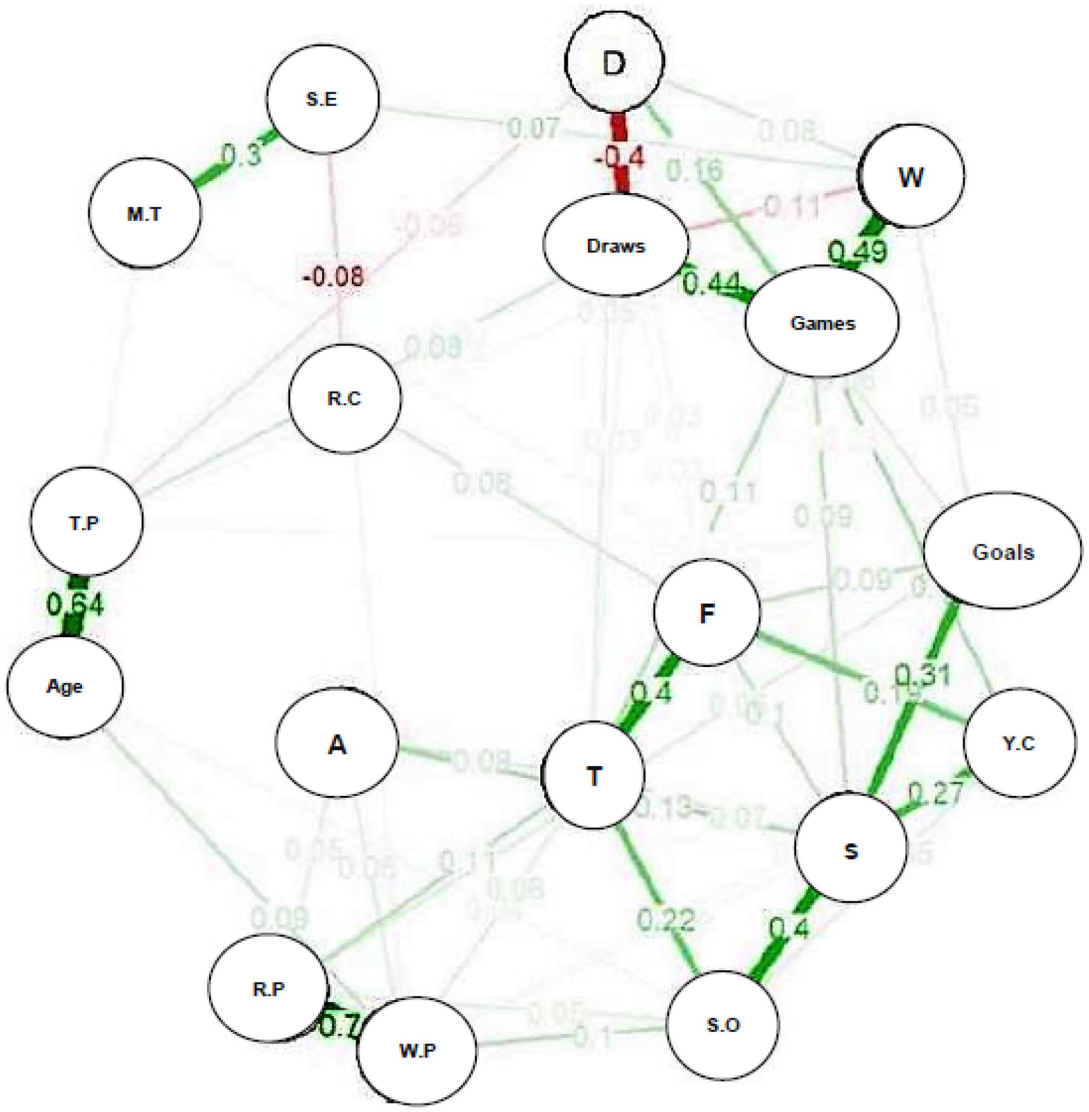
Perceived sport self-efficacy, mental toughness and performance data of the NFL 2020 teams from Paraná (*γ* = 0.25; *n* = 77). A, assists; D, defeats; F, fouls; M.T, mental toughness; R.C, red cards; R.P, right passes; S.E, self-efficacy; S.O, shots out; S, shots; T.P, time of practice; T, tackles; W.P, wrong passes; W, wins; Y.C, yellow cards.

In Figure 1, the analyzed model brings a greater balance between specificity and sparsity, with the hyperparameter gamma (*γ* = 0.25), in which the spatial distribution of the variables is observed, one of the indicators of their interrelationships. Age, time of practice and psychological characteristics were positioned at the upper left end of the network (close to red cards), while the number of games and their results (win, draw, or defeat) were grouped on the right side.

In terms of connections, we highlight some of the relationships that connect the different groupings of the network. The Perceived Self-Efficacy Scale in Sports (PSES) showed a positive relationship with mental toughness (MT) (*r* = 0.30), playing its role indirectly, through this connection. In addition, PSES showed connections with the number of wins (*r* = 0.07), a negative relationship with the number of red cards (*r* = −0.08) and wrong passes (*r* = −0.06); time of practice had an inverse relationship with the number of defeats (*r* = −0.06) and age was positively related to the number of wrong passes (*r* = 0.09) (Figure 1).

**Figure 2:**
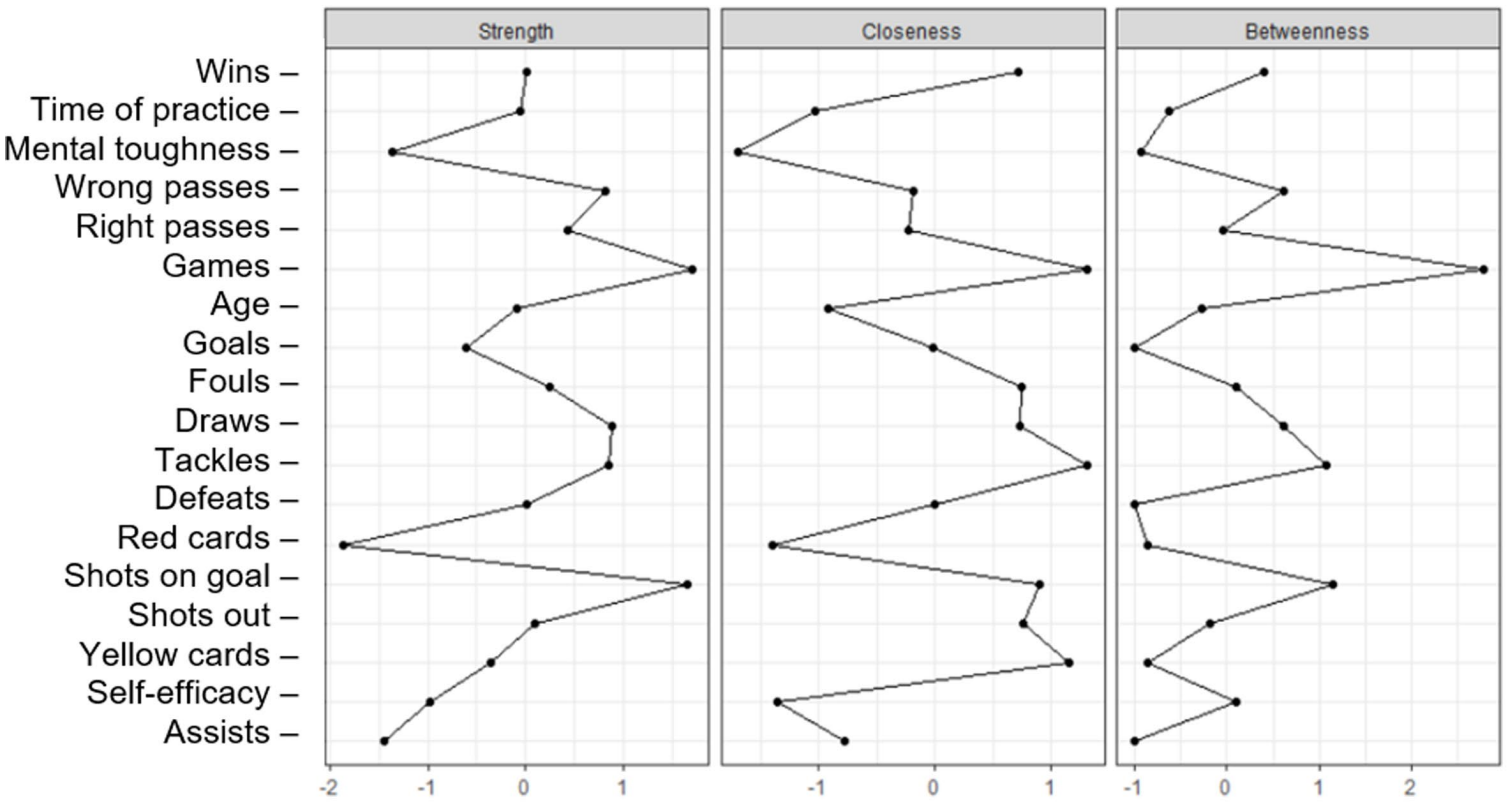
Network centrality indicators (NCI) of perceived sport self-efficacy, mental toughness and performance data of NFL 2020 teams from Paraná (*γ* = 0.25; *n* = 77).

Figure 2 presents the Network Centrality Indicators (NCI).

The number of games played proved to be the most central variable in the network, due to its degree of strength, proximity and intermediation. The high degree of proximity and intermediation of the tackles made stands out, which was connected to the number of assists (*r* = 0.08). All correlations were significant (*p* < 0.05).

## Discussion

The objective of this study was to examine the impact of Perceived Self-Efficacy Scale in Sports (PSES) mediated by mental toughness (MT) on sport performance in Brazilian futsal athletes. This is the first study to relate PSES, MT and performance in Brazilian futsal athletes using a specific instrument to investigate PSES.

In Table 1, it was possible to observe that the investigated sample presented high levels of PSES and MT. The best ranked teams (teams 1, 2, and 3) presented higher amounts of goals for, fouls, shots on goal, tackles, assists, and wins. It is noteworthy that futsal is an extremely dynamic sport, with extremely fast and constant motor actions, as well as the exchange of positions among athletes, especially during the offensive phase (Müller et al., 2018; Bueno et al., 2020).

The teams with the greatest number of goals, shots on goal and best final ranking also presented the greatest number of tackles, fouls, yellow cards and assists. This fact can be explained by the specificity of this modality, in which many tackles are made near the opponent’s goal, which may result directly in a shot on goal or even an assist. Nevertheless, these technical gestures often end up generating a foul for the opposing team (Pizarro et al., 2020).

Borges et al. (2021) stated that futsal demands a great deal of energy from its athletes, both in the physical and mental aspects. Because it is an extremely dynamic sport, it is important that the athletes have a good PSES, as well as MT, as our findings showed. The mental skills of the athletes stand out when we observe that, even with a considerable amount of fouls, such penalties, generate few expulsions and, at the same time, create good offensive actions for the teams, showing that the athletes have good mental control and good decision-making in the choice of the sportive technical gesture, fundamental for a good athletic performance (Spyrou et al., 2020) (Table 1).

In the network analyses, the importance of the connections established by self-efficacy (SE) stands out. PSES showed a positive relationship with the number of wins, and negative relationships with the number of red cards and wrong passes. Mental toughness (MT) indirectly influenced these variables through its connection with PSES (Figure 1).

This shows that these athletes can obtain good levels of performance, through good relationships with the wins and fewer wrong passes. In addition, they have emotional and technical control to avoid expulsions. According to Uchida et al. (2018) Perceived Self-Efficacy Scale in Sports (PSES), relates to the individual’s ability to self-regulate, due to his/her ability to control and modulate his/her behavior to achieve his/her goals and objectives, in addition to presenting better self-control and performance skills (Du and Zhang, 2022). Such findings are in line with recent research considering that high levels of SE are positively associated with success and better sport performance (SP) (Ayazi et al., 2013; Butt et al., 2017; Weight et al., 2020; Li et al., 2022).

The time of practice showed an inverse relationship with the number of defeats, while age remained positively related to the number of wrong passes (Figure 1). According to Aizava et al. (2021), athletes with longer experience in the sport tend to present a higher PSES, obtaining a better sport performance, which may explain the negative relationship with defeats (Arribas-Galarraga et al., 2020; Machado et al., 2021). However, in an investigation with psychological variables in women cyclists, Abenza-Cano et al. (2021) did not observe a significant relationship between age, time of practice and the performance of the athletes.

For Norheim et al. (2020), with advancing age, there is a gradual decrease in muscle strength and muscle power, especially in the lower limbs, resulting in a critical role in terms of determining the competitiveness and performance of athletes. Rey et al. (2022) found that players over 30 years of age show significantly lower performance in total traveled distance, high-intensity activities, sprint distance and number of accelerations and decelerations. However, there are few investigations about the alterations in the technical and tactical performance of athletes in relation to their chronological age. In this regard, it is observed that, for older athletes, some specific technical gestures may become more difficult due to the aging process itself, which brings physiological and physical alterations to individuals, influencing the quality of motor task execution (Lorenzo-Martínez et al., 2021; Borghi-Ricardo et al., 2022).

Our findings allow us to state that, despite the fact that older athletes present more passing errors, they are able to obtain better sporting success, due to the good relationship with the number of wins. According to Ayazi et al. (2013), this can be explained by the fact that PSES is inversely related to the fear of success, that is, athletes try to perform more motor actions, even more complex ones, becoming more susceptible to errors, but obtaining positive results in the end (Butt et al., 2017; Weight et al., 2020).

In this regard, the results allowed us to identify that having a tough mindset is important for the athlete to feel more self-effective, leading athletes to practical behaviors and results, due to the intermediation done by PSES in the connection between MT and other nodes in the network. According to Brace et al. (2020), judgments carried out by SE in the context of MT involve complex processes of self-evaluations and persistence in extenuating circumstances.

Although still understudied, these factors are related to and influence the performance of other sport behaviors, and should be understood as shared attributes. Rintaugu et al. (2022) stated that having a tough mindset means being mentally strong, and that the athlete has acquired thinking, believing and visualizing skills that provide empowering emotions during competitions (Zeiger and Zeiger, 2018; Ramolale et al., 2021), in addition to showing better career development capacity, pressure resistance, training and good performance (Tian et al., 2022).

In study of Meggs et al. (2014), it was possible to identify, through self-report measures in 105 British athletes from different modalities (wrestling, swimming, dance, athletics, tennis, and rowing) in national level competitions, that MT was positively associated with psychological variables such as self-concept, and was particularly high in individuals with goal commitment, despite obstacles and the potential for failure. This reveals important implications for how beliefs about efficacy and MT interact and affect positive and pro-social behavior in sports. MT is an essential characteristic of the best athletes, who have better mental resources during performance sports (Kosirnik et al., 2022) and can be developed and improved over time, especially when there is a good relationship between coaches and athletes (Caruzzo et al., 2021).

We should also highlight the difference between the red cards and the other variables in the networks (Figure 1), due to its more isolated positioning, without establishing connections with the number of yellow cards, and with greater proximity to the psychological characteristics. This shows that athletes with good levels of SE and MT present imminent psychological characteristics in relation to the futsal game. They make many tackles during the matches, without making too many violent fouls or even pointing out emotional imbalances, which could result in more expulsions (Bueno et al., 2020; Pizarro et al., 2020; Spyrou et al., 2020; Borges et al., 2021).

In a study of elite figure skaters, Barkhoff and Heiby (2010) stated that PSES is directly linked to levels of competition. Their research showed that medal winning athletes (with better performance) revealed higher levels of PSES. It is noteworthy that, according to Han et al. (2022) regularly physically active individuals tend to have better PSES, as well as good mental health, important factors in SP. Nevertheless, Rogowska et al. (2022) identified that PSES more than simply is positively related to SP, but rather a predictor/mediator of performance.

The NCI (Figure 2) showed that the number of games played is the variable with the highest degree of power (strength) and intermediation (betweenness), and the second with the highest in degree of proximity (closeness). Tackling was the variable with the highest degree of closeness and the second with highest degree of intermediation, explaining its more centralized positioning in the networks.

Pizarro et al. (2020) emphasized the importance of defensive actions in futsal. Among them, tackling is one of the most important fundamentals. Our findings allowed us to show its importance for the other aspects of the game, by revealing connections with fouls, shots and the number of right passes, due to the specificity of this sport in which many times, when tackling, the athlete seeks an offensive pass, shots on goal, but it is an action that can result in a foul for the opponent if executed incorrectly (Bueno et al., 2020; Spyrou et al., 2020; Borges et al., 2021). The good levels of MT found are in line with the study of Rintaugu et al. (2022), in which the authors emphasized the importance of this variable in some team sports (soccer and handball), and that these tend to provide good MT to the athletes.

The current research presented some limitations regarding the sample size, which may be the main responsible for the disappearance of some connections as the parsimony of the network was increased, indicating that the observed relationships deserve to be tested again in future studies with a larger number of observations to feed the network. In addition, the difficulties imposed by the COVID-19 pandemic (changes in the research project, target population, championship regulations, access to teams) certainly constituted a limiting factor for the development of the research.

## Conclusion

The findings of the present research allowed us to observe that the investigated athletes of the NFL 2020 teams presented good levels of self-efficacy and mental toughness. The investigated teams performed well, with all teams staying in the league and some advancing to the final stages. Until this moment, all investigated teams remain in the main national futsal league in Brazil.

Through the network analysis, it was possible to note a good relationship between Perceived Self-Efficacy Scale in Sports (PSES) and mental toughness (MT), in which having a tough mindset seems to be important for a better PSES, mainly because they have shown to be intervening factors in variables such as tackling, passes and shots on goal, which are fundamentals for a good performance in futsal, due to the intermediation done by PSES in the connection between MT and other nodes of the network. Nonetheless, it is noteworthy that more experienced athletes feel more effective than younger athletes.

As practical implications, we hope that the present study can be used by sport psychologists and coaches, as well as athletes, as a way of optimizing the processes of training, competitions, and even sport performance. Inducing positive emotions related to great achievements and strategies for coping with failure are important elements in this context. Increasing and enhancing perceptions of PSES and MT are essential to achieve good sport performance, specifically for athletes in team sports such as futsal.

## Data availability statement

The raw data supporting the conclusions of this article will be made available by the authors, without undue reservation.

## Ethics statement

The studies involving human participants were reviewed and approved by Standing Committee on Ethics in Research with Human Beings—Maringá State University (Opinion no 4.022.246). The patients/participants provided their written informed consent to participate in this study.

## Author contributions

All authors listed have made a substantial, direct, and intellectual contribution to the work and approved it for publication.

## Funding

This study was financed in part by the Coordenação de Aperfeiçoamento de Pessoal de Nível Superior—Brasil (CAPES)—Finance Code 001.

## Conflict of interest

The authors declare that the research was conducted in the absence of any commercial or financial relationships that could be construed as a potential conflict of interest.

## Publisher’s note

All claims expressed in this article are solely those of the authors and do not necessarily represent those of their affiliated organizations, or those of the publisher, the editors and the reviewers. Any product that may be evaluated in this article, or claim that may be made by its manufacturer, is not guaranteed or endorsed by the publisher.
